# Determination of carriers of deafness-infertility syndrome in Peru

**DOI:** 10.1186/s13023-025-04002-w

**Published:** 2025-11-18

**Authors:** Luana Carolina Ventura Cuellar, Kevin Bryan Aviles Jascha, Hugo Hernan Abarca-Barriga

**Affiliations:** 1 Universidad Cientifica del Sur, Lima, Peru; 2 Instituto Nacional del Niño, Breña, Lima, Peru

**Keywords:** CATSPER2, STRC, Male infertility, Deafness, CNV

## Abstract

The prevalence of deafness-infertility syndrome (DIS) is approximately 1%. Genetic heterogeneity is one cause of homozygous copy number variants (CNVs) involving the *CATSPER2* and *STRC* genes, which are associated with DIS and male infertility. Because the prevalence of DIS in Peru is unknown, we aimed to determine the frequency of carriers of DIS-related genes. In this descriptive crossover study, we evaluated the clinical histories and chromosomal microarray analysis results of patients at the Instituto Nacional de Salud del Niño Breña from 2015 to 2022. All patients with CNVs involving the CATSPER2 and STRC genes were included, and the frequencies of carriers and affected patients were determined using Hardy‒Weinberg equilibrium. Relative frequency differences were calculated using the chi-square test with goodness-of-fit for natural regions and poverty groups in Peru. Of 2,142 patients screened, 35 met the inclusion criteria; according to the results, approximately 367,364 people were estimated to be DIS carriers in Peru, and approximately 57,442 people had deafness and infertility. The proportion of carriers in Peru was similar to that observed in other population studies. Additionally, people in regions with higher poverty rates exhibited a greater carrier frequency, suggesting that a patient’s region of origin could be a criterion for DIS screening.

## Introduction

Overall, 6 to 8% of the population has a rare disease, and 80% of these diseases have a genetic origin [1]. Similarly, it is estimated that 24% of people are carriers of at least one recessive disease (X-linked or autosomal) [2]. In addition, the frequency of copy number variations (CNVs) in DNA is at least 2% [3]. Futhermore, it is estimated that people have at least one heterozygous variant of an autosomal recessive disease, and on average, they can carry between 2 and 5 variants of autosomal recessive diseases [4]. Hearing loss causes greater learning difficulties, resulting in a higher incidence of poverty and lack of employment in adulthood [5]. Similarly, infertility is a common factor in psychological illnesses that could impact the economic sector [6].

Among these rare diseases, deafness-infertility syndrome(DIS) is characterized by a CNV of chromosome 15q15.3, in loss (homozygous deletion) at the DFNB16 locus, involving the *CATSPER2* and *STRC* genes; which is characterized by asthenotherozoospermia (males), and prelingual sensorineural hearing loss (both sexes) of mild to moderate intensity [7–9]. In China, this syndrome was reported in three consanguineous families [9] and in France, the United States, Israel and the Netherlands, patients were reported in non-consanguineous families [7, 8, 10]. These previous findings suggest the importance of conducting studies in other populations to understand the importance of consanguinity in rare diseases.

Variants in the *STRC* gene results in syndromic sensorineural hearing loss (SNHL), due to the fact that some variants are associated with the *CATSPER* gene, while variants in the *GJB2* gene are mainly related to nonsyndromic SNHL [11]. It is estimated that 2 out of every 1000 newborns are affected with sensorineural hearing loss (SNHL), 50–70% of these cases are of genetic origin, 75% of which have autosomal recessive inheritance.

Variants in the *STRC* gene are linked to 1–5% of patients with mild to moderate SNHL [12]. On the other hand, as SNHL is an under-diagnosed condition, a routine genetic test can be implemented as a suggestion to achieve a differential diagnosis in those cases of congenital hearing impairment [13] which allows a new public health criterion for premarital screening to be proposed as a preventative approach.

The *CATSPER2* gene, which is involved in “capacitation, hyperactivation, acrosomal reaction and sperm motility” [12, 14], has been linked to male infertility [9]. Therefore, homozygous deletion of *CATSPER2* can cause asthenoteratozoospermia [14], resulting in a decreased penetrative ability, impaired hyperactivation and the absence of response to progesterone [7]. Regarding the prevalence of deafness-infertility syndrome, few studies have been performed at the global and national scale [7–9, 12, 14, 15].

Being the aim of the study to determine the frequency of carriers DIS syndrome in Peru, based on the patients attended at the INSN, which is one of the most important national reference centers in pediatric care and the only institution that performs this genetic test. The results of this study could identify the region with the highest frequency of DIS; this knowledge can be applied at the public health level to raise awareness of the necessity of premarital genetic testing.

## Methods

### Study design

A cross-sectional study was performed in which patients who underwent chromosomal microarray analysis (CMA) at the National Institute of Child Health from 2015 to 2022 were included.

### Study population

The study population was defined by the following selection criteria: patients who underwent CMA from 2015 to 2022 with quality indices above those stipulated by Affymetrix ^®^. Patients with medical records that were incompletely digitized in the database and those whose parents or guardians did not allow their participation were excluded from the study.

### Sample

The medical records composed of all patients with DIS carriers from 2015 to 2022 found in the database of the Genetics & Inborn Errors of Metabolism (IEM) Service of the Instituto Nacional Del Niño were selected. For sample selection, non-probability convenience sampling was applied, with a statistical power of 80% with 95% CI.

### Study variables

For DIS carriers, patients with deletion of *CATSPER2* and *STRC* genes were included [7]. Both sexes were included, aged 0 to 18 years; who underwent the CMA test, with diagnoses such as neurodevelopmental disorders (intellectual disability, autism spectrum disorder, psychomotor developmental delay) and others (language delay, short stature, congenital anomalies, etc.). In addition, information such as parental age, CNV size, and uniparental disomies and parental consanguinity were extracted by homozygosity regions (ROH).

Likewise, patients, parents and grandparents were classified according to dsitricts and regions of origin. Additionally, to analyze the demographic characteristics of the participants, we classified them into poverty groups by considering the region of origin of the patients’ grandparents as a reference to ensure greater representativeness of our study, taking as a reference the technical report of the evolution of monetary poverty in the INEI for the year 2022 [16, 17].

On the other hand, the Hardy Weinberg equilibrium states that allelic and genotypic frequencies must remain constant throughout the generations of a population in order to maintain balance. For this to be fulfilled, the population must be infinite, there must be no individuals from another population, mating must be random, i.e., there must be no natural selection, and there must be no genotypes more effective than others [18].

### Bias

Although the ideal approach is to study the general population, the INSN is a national reference institute for performing CMA in patients with diagnoses different from that of interest to our study, where the identification of a DIS carrier is incidental or secondary, which would allow extrapolation of our results to the general population. Furthermore, due to the number of cases found, further extension of the analysis was not possible. On the other hand, as this study is based on medical records (secondary sources), information bias may be present; thus, some data may have recording and digitization errors.

### Procedure

CMA was performed according to the GeneChip CytoScan 750 K Array protocol (Affymetrix, USA ^®^) following the manufacturer’s instruction manual. The test includes 550 000 non-polymorphic markers and 200 436 markers with single nucleotide polymorphism. The cells in rows are scanned using the Chromosome Analysis Suite (ChAS) software (Affymetrix, USA ^®^). For gains or losses are due to the compromise of at least 50/25 markers respectively. In ROHs, a length of at least 10 Mpb is impaired [19].

### Statistical methods

#### Descriptive analysis

For quantitative variables, a normal distribution was obtained, to then establish the mean and standard deviation (parents’ age). On the other hand, a non-normal distribution was used to establish median and interquartile range (patient age).

Absolute and relative frequencies were calculated for qualitative variables.

By applying the Hardy-Weinberg law, the possible frequency of carriers in the Peruvian population was estimated assuming the following premises: absence of migration, infinite population, absence of genetic drift, no natural selection, random reproduction and one genotype should not be more effective than another; applying the following formula:

$${\left( {p + q} \right)^2} = {p^2} + 2pq + {q^2}$$

where p^2^: homozygous dominant.

2pq: heterozygous.

q^2^: recessive homozygote.

Thus, based on a total number of participants who underwent the CMA in this study, and data from the Peruvian population according to INEI. The formula was applied to estimate healthy, sick and carrier patients, upper and lower limits were estimated with a 95% CI [20, 21].

#### Bivariate analysis

The chi-square test with goodness of fit was used to verify whether there were differences in the proportions of DIS and *CATSPER2* gene deletion carriers according to natural region or origin and poverty group according to the most recent INEI report. Patients were divided into poverty groups: Group 1 corresponded to the regions of Cajamarca; Group 2 corresponded to Amazonas, Ayacucho, Huancavelica, Huánuco, Loreto, Pasco and Puno; Group 3 corresponded to Ancash, Apurimac, Junín, La Libertad, Lima, Moquegua, Piura, Callao, and San Martin; Group 4 corresponded to Arequipa, Cusco, Lambayeque, Metropolitan Lima, Madre de Dios, Tacna, Ucayali; Group 5 corresponded to Ica and Tumbes [16, 17].

Statistical significance was defined as *p* < 0.05 and a confidence level of 95%. The following statistical programs were used: STATA version 15.0 and Jamovi version 2.3.

### Ethical aspects

The present study complied with ethical research considerations: the data were used anonymously, personal information such as email address was not requested, and patient identity was anonymized; therefore, informed consent was not required. The information used in the study was obtained with the consent of the respective authorities of the Instituto Nacional Del Niño (institutional and ethical approvals, number N°237-2022-CIEI-INSN). Likewise, the Ethics Committee of the Universidad Cientifica Del Sur approved this project prior to its implementation (Registration number: PRE-15-2022-00459).

## Results

In the present study, out of 2,142 patients who underwent CMA, 35 patients with variants were included; 26 patients had variants involving the *STRC* and *CATSPER2* genes, and 9 patients harbored deletion of the *CATSPER2* gene. The median age of the children evaluated was 4 years (RIC 3–6), the average age of the fathers was 39 years and this difference is 5 years greater than the age of the mother. There is a difference in the proportion of gene affectation only by *CATSPER2* and those presenting *CASTSPER2* and *STRC* (*p* = 0,004). At the level of natural region of origin, a clear difference is shown in those who are from the coast in relation to the rest of the natural regions (*p* < 0,05). The 54.3% of the cases found are female and 68.6% have no consanguinity according to the CMA (ROH < 0,05). Finally, the patients underwent the CMA test whose main diagnosis was neurodevelopmental disorders 71.4% (Table 1).

**Table 1:** Epidemiological data of children who underwent chromosomal microarray analysis at INSN

Variable	*n*	%	*p*
**Age**			
Children(median/IQR)	4	(3–6)	
Mother*	34.0	10.1	
Father*	39.1	12.2	
**CNV size**			
**Minimum coordinate**			
Minimum value	43,553,262		
Maximum value	43,636,120		
**Maximum coordinate**			
Minimum value	43,684,983		
Maximum value	43,715,339		
**Sex**			
Male	16	45.7	0.612
Female	19	54.3	
**Consanguinity (ROH > 0.5)**			
Yes	11	31.4	0.028^£^
No	24	68.6	
**Gene involvement**			
*CATSPER2*	9	25.7	0.004^£^
*CATSPER2 y STRC*	26	74.3	
**Region of origin**			
**Region of child**			
Coast	25	71.4	0.067
Highlands	6	17.1	0.062
Rainforest	1	2.9	0.061
Foreigners	3	8.6	-
**Mother’s region**			
Coast	18	51.4	0.005^£^
Highlands	11	31.4	0.564
Rainforest	3	8.6	0.319
Foreigners	3	8.6	-
**Father’s region**			
Coast	23	65.7	0.035^£^
Highlands	10	28.6	0.411
Rainforest	1	2.9	0.061
Foreigners	1	2.9	-
**Maternal grandfather’s region**			
Coast	19	54.3	0.007^£^
Highlands	13	37.1	0.917
Rainforest	0	0	0.020
Foreigners	3	8.6	-
**Maternal grandmother’s region**			
Coast	17	48.6	0.003^£^
Highlands	14	40	0.901
Rainforest	1	2.9	0.061
Foreigners	3	8.6	-
**Paternal grandfather’s region**			
Coast	21	60	0.008^£^
Highlands	12	34.3	0.602
Rainforest	1	2.9	0.050
Foreigners	1	2.9	-
**Paternal grandmother’s region**			
Coast	21	60	0.008^£^
Highlands	11	31.4	0.449
Rainforest	2	5.7	0.127
Foreigners	1	2.9	-
**Diagnostics**			
neurodevelopmental disorders	25	71.4	
others	10	28.6	

Neurodevelopmental disorders: intellectual disability, autism spectrum disorder and psychomotor developmental delay. Others: language delay, short stature, congenital anomalies, etc

IQR: interquartile range. SD: standard deviation. CNV: copy number variants

*SD and mean were calculated. ^£^*p*-value according to chi-square with goodness of fit according to the actual proportions of the different regions based on INEI data. Coast: 55.9%, highlands: 29.6% and jungle: 14.5%

Of a total of 26 patients with DIS, it was found that the origin of the patients with DIS according to departments at the national level was Lima 64.0% (*n* = 14) and Junín 8.0% (*n* = 2). However, three patients were found to be foreigners and were not included in the analysis. Of a total of 104 maternal and paternal grandparents of the DIS carriers, where 8 subjects of foreign origin were excluded, it was found that the most frequent origins of the grandparents were from Lima 38.5% (*n* = 37), Junín 12.5% (*n* = 12) and Cajamarca 9.4% (*n* = 9). Among the 9 patients with the *CATSPER2* deletion, 100% were from Lima. Meanwhile, of a total of 36 maternal and paternal grandparents, it was found that the most frequent origins of the grandparents were from Lima 22.2% (*n* = 8), Piura 22.2% (*n* = 8) and Ayacucho 19.4% (*n* = 7) (Table 2).

**Table 2:** Districts of origin of patients carrying DIS and maternal and paternal grandparents

Districts	Districts of origin of carrier patients				Districts of origin of maternal and paternal grandparents			
	DIS	%	CATSPER2	%	DIS	%	CATSPER2	%
Amazonas	0	0.0	0	0.0	0	0.0	0	0.0
Áncash	0	0.0	0	0.0	5	5.2	0	0.0
Apurímac	0	0.0	0	0.0	0	0.0	0	0.0
Arequipa	0	0.0	0	0.0	4	4.2	0	0.0
Ayacucho	1	4.0	0	0.0	6	6.3	7	19.4
Cajamarca	1	4.0	0	0.0	9	9.4	5	13.9
Callao	0	0.0	0	0.0	0	0.0	0	0.0
Cusco	0	0.0	0	0.0	2	2.1	0	0.0
Huancavelica	0	0.0	0	0.0	4	4.2	0	0.0
Huánuco	1	4.0	0	0.0	2	2.1	1	2.8
Ica	0	0.0	0	0.0	0	0.0	0	0.0
Junín	2	8.0	0	0.0	12	12.5	4	11.1
La Libertad	1	4.0	0	0.0	6	6.3	2	5.6
Lambayeque	1	4.0	0	0.0	1	1.0	0	0.0
Lima	14	64.0	9	100.0	37	38.5	8	22.2
Loreto	0	0.0	0	0.0	2	2.1	0	0.0
Madre de Dios	0	0.0	0	0.0	0	0.0	0	0.0
Moquegua	0	0.0	0	0.0	0	0.0	0	0.0
Pasco	0	0.0	0	0.0	0	0.0	0	0.0
Piura	1	4.0	0	0.0	4	4.2	8	22.2
Puno	0	0.0	0	0.0	0	0.0	0	0.0
San Martín	0	0.0	0	0.0	1	1.0	1	2.8
Tacna	0	0.0	0	0.0	0	0.0	0	0.0
Tumbes	1	4.0	0	0.0	1	1.0	0	0.0
Ucayali	0	0.0	0	0.0	0	0.0	0	0.0
Total	23	100.0	9	100	96	100.0	36	100.0

*Chi-square test with goodness of fit according to the actual proportions of the different districts based on INEI data^£^

Of a total of 104 maternal and paternal grandparents of the DIS carriers, where eight subjects of foreign origin were excluded (*n* = 96), the highest frequency of DIS carriers according to the poverty group established by the INEI was found in group 1 with 9.4% (*n* = 9) coming from the department of Cajamarca; being the only group with significant differences (*p* = 0.020). Regarding the maternal and paternal grandparents of the carriers of the *CATSPER2* deletion, out of a total of 36, the highest frequency of grandparents of carriers with respect to the poverty group is found in group 3, which includes Lima; however, only groups 1, 2 and 4 present significant differences with the values expected by the INEI (*p* < 0,05). In both analyses, the frequency of foreigners was not considered because there is no report of the proportion of foreigners in the Peruvian population (Table 3).

**Table 3:** Districts groups with poverty groups of grandparents

Poverty groups	Frequency of DIS carriers	%	p*	Carrier frequency (deletion in CATSPER2)	%	p*
Group 1^£^ (4,48%)	9	9,38%	0,020	5	13,89%	0,006
Group 2^£^ (11,64%)	14	14,58%	0,369	8	22,22%	0,048
Group 3^£^ (32,24%)	35	36,46%	0,377	16	44,44%	0,117
Group 4^£^ (47,69%)	37	38,54%	0,073	7	19,44%	< 0,001
Group 5^£^ (3,95%)	1	1,04%	0,143	0	0%	0,224
TOTAL	96	100,0%		36	100,0%	

* *p*-value according to chi-square with goodness of fit according to the actual proportions of the different poverty groups based on INEI data^£^

Group 1: Cajamarca

Group 2: Amazonas, Ayacucho, Huancavelica, Huánuco, Loreto, Pasco, Puno

Group 3: Ancash, Apurimac, Junin, La Libertad, Lima, Moquegua, Piura, Const. Prov. del Callao, San Martin

Group 4: Arequipa, Cusco, Lambayeque, Metropolitan Lima, Madre de Dios, Tacna, Ucayali

Group 5: Ica and Tumbes

Based on the Hardy-Weinberg equilibrium with a total population of 33 millions 396,700 inhabitants, it was found that 36,364 inhabitants would be carriers of DIS, with a frequency of 1.1%. While, the affected persons would range from 1 to 57,442 Peruvian population (CI 95%) (Table 4).

**Table 4:** Frequency and confidence interval for the DIS in the Peruvian population

DIS	*n*	%	Frequency according population	Confidence interval at 95%.	
*N*°				Lower	Upper
Healthy	2119	98,9	33 029 336	32 860 015	33 168 935
Carriers	23	1,1	367 363	227 765	536 685
Sick	0	< 0,001	< 0,001	0	57 442
Total	2142	100%	33 millones 396 mil 700 habitants		

By evaluating only the *CATSPER2* gene deletion, and applying again the Hardy-Weinberg equilibrium, it was found that 133,587 Peruvian inhabitants would have a heterozygous deletion in Peru, with a frequency of 0.4% (Table 5).

**Table 5:** Frequency and confidence interval for CATSPER2 in the Peruvian population

CATSPER2	*n*	%	Frequency according population	Confidence interval at 95%.	
*N*°				Lower	Upper
Healthy	2133	99,6	33 263 113	33 130 862	33 332 578
Carriers	9	0,4	133 587	6 412	265 838
Sick	0	< 0,001	< 0,001	0	57 442
Total	2142	100%	33 millones 396 mil 700 habitants		

## Discussion

In the present study, 26 patients with heterozygous deletions in the *CATSPER2* and *STRC* genes were reported; and based on the Hardy-Weinberg equilibrium, it was estimated that 1.1% of the national population could be carriers (367,363 Peruvian population). In a previous study in United States, a frequency of 1% DIS carriers is proposed, with a prevalence of 1 in 40,000 people [9]; this value is similar to the frequency found in this study. On the other hand, a national study (*n* = 400) reported 12 children with heterozygous deletions of DIS, where 3 out of every 100 people could carry this condition; however, the data may have been overestimated because the study was performed in a minor population [19].

A systematic review and meta-analysis estimated that 1.36% of a population without hearing problems could be autosomal recessive carriers for *STRC* variants [22]. In addition, it has been described that non-syndromic hearing loss is more frequently autosomal recessive, where approximately 50% involve the *DFNB1* locus, caused by variants in the *GJB2* and *GJB6* genes [9]. The second most frequent cause of mild to moderate hearing loss belonged to homozygous deletions in *STRC* [12]. However, no CNVs involving only the STRC gene were found to contrast our findings with data from other investigations.

On the other hand, nine patients with deletions in the *CATSPER2* gene were found. Likewise, it was estimated that the possible frequency of carriers according to Hardy-Weinberg’s equilibrium was 0.4% at national level, with a possible affection from 1 to 133,587 Peruvian inhabitants carrying this deletion. Currently, no frequencies have been found for the *CATSPER2* gene, and worldwide there are studies where the *CATSPER2* deletion is linked to the *STRC* deletion and the detection is secondary to the analysis of the *STRC* gene [22, 23]. Similarly, another study indicates that its detection is currently not possible through the application of clinical tests and for this reason it is underdiagnosed as a causal factor of male infertility [14].

One of the limitations of the present study is its cross-sectional nature, which does not allow us to establish a cause-effect relationship of the associated variables. In addition, although it would be ideal to study the general population, the INSN is a national reference institute for the performance of CMA in patients with diagnoses other than the disease of interest in our study; therefore, the identification of a DIS carrier is incidental or secondary, which could allow for an extrapolation of the results to the general population. Furthermore, due to the number of available cases, it was not possible to extend the analysis. In addition, as this study is based on medical records (secondary sources), there may be information bias; therefore, some data may have recording and digitization errors. Finally, we attempted to characterize the poverty levels of the families in the study, although it would have been ideal to obtain information on the origin of the grandparents at the district level for the best approximation of poverty. When applying Hardy-Weinberg equilibrium, the results would be an estimate but would not necessarily reflect the true value of the carrier frequency because the assumptions would not be fully met, such as migratory movements or non-random matching.

Finally, it was observed that the DIS carriers and their grandparents probably come from Lima and Junín. Meanwhile, *CATSPER2* deletion carriers and their grandparents come mainly from Lima. Regarding the poverty groups, a higher frequency of DIS carriers was found in those families that came from group 1 and *CATSPER2* in those families that came from groups 1 and 2, who are also in a group with a higher incidence of poverty at the national level according to the INEI, and show a p-significance [16, 17]; however, there were no other studies that could provide information on these data.

## Conclusions

In this study, we report that the frequency of DIS carriers is similar to that reported in the United States, but there are no concise frequencies in Europe, Asia and Africa. Additionally, in the departments where we found a higher frequency of carriers was in Lima and Junín. Concerning the poverty group, those who belonged to group 1 would have a great possibility of having a higher frequency of DIS carriers.

The findings of this study could be a basis for new studies focusing on the importance of early diagnosis to reduce the impact of DIS on public health. Further research might be required to investigate the etiologies and various treatments in premarital or preconceptional counseling.

### Recommedations

Due to the likelihood of finding a higher frequency than expected, it is essential to perform genetic studies such as multiplex PCR or multiplex ligand dependent probe amplification (MLPA) for the diagnosis of this condition. Thus, a prevalence study would allow early and timely management in terms of clinical decision making; with respect to the application in health management, it will allow proposing a new public health criterion starting from a preventive approach to premarital screening. On the other hand, as hearing loss is underdiagnosed, it should be considered as an initial screening for DIS and should be included in the differential diagnosis of people with hearing loss.

## Data Availability

The data supporting the findings of this study are available via the Universidad Cientifica del Sur online data repository: https://hdl.handle.net/20.500.12805/3440.

## Abbreviations

IEM: Genetics & Inborn Errors of Metabolism
ChAS: Chromosome Analysis Suite
